# Outbreak Investigation of a Multipathogen Foodborne Disease in a Training Institute in Rabat, Morocco: Case-Control Study

**DOI:** 10.2196/14227

**Published:** 2019-09-25

**Authors:** Houda Moumni Abdou, Ilham Dahbi, Mohammed Akrim, Fatima Zahra Meski, Yousef Khader, Mohammed Lakranbi, Hind Ezzine, Asmae Khattabi

**Affiliations:** 1 Field Epidemiology Training Program Ecole Nationale de Santé Publique Ministry of Health Rabat Morocco; 2 Department of Community Medicine, Public Health and Family Medicine Faculty of Medicine Jordan University of Science & Technology Amman Jordan; 3 Directorate of Epidemiology and Disease Control Ministry of Health Rabat Morocco; 4 Laboratoire “Biodiversité, Ecologie et Génome” Faculté des Sciences Université Mohammed V Rabat Morocco

**Keywords:** disease outbreaks, foodborne diseases, Staphylococcus, Escherichia coli

## Abstract

**Background:**

On June 18, 2017, the public health service was alerted about 43 students in the training institute in Rabat who were admitted to the emergency room for acute gastroenteritis following the uptake of a meal a day before.

**Objective:**

This study aimed to investigate the foodborne disease outbreak by confirming the outbreak, identifying the source of contamination, and recommending control measures.

**Methods:**

We conducted a case-control study. Cases and controls were selected in a ratio of 1:1. We defined a case as any member of the training institute who attended the Ramadan buffet in the institute’s restaurant and who had presented, in the weekend of June 16 to 20, 2017, symptoms of diarrhea or vomiting with at least one of the following signs: abdominal pain, fever, headache, nausea, and dizziness. A control was defined as anyone who attended the Ramadan buffet in the institute’s restaurant but had not presented any symptoms from June 16 to 20, 2017. We conducted a bivariate and multivariable analysis. Stools of ill students were collected, and a food specimen was collected for bacterial testing.

**Results:**

A total of 50 cases and 50 controls were selected. Among the cases, males were predominant (43/50, 86%); the median age was 21 years. A total of 47 cases sought medical care. There were no hospitalizations and no deaths. The episode was short with an estimated average incubation period of 9 hours. The epidemic curve oriented toward a common source of contamination. Among food items, briwates were strongly associated with the illness with an odd ratio of 14.23 (95% CI 5.04-40.04; *P*<.001). Laboratory testing of briwates showed presence of *Escherichia coli* O157 and *Staphylococcus aureus*.

**Conclusions:**

This foodborne disease outbreak was likely caused by briwates that was contaminated with *S aureus* and *E coli*. We recommended strengthening hygiene measures. Food handling techniques should be taught as part of continuous professional development for food handlers.

## Introduction

Acute collective food poisoning or foodborne disease outbreak (FBDO) occurs when 2 or more people develop a similar illness after ingesting the same contaminated food or drink [1]. Illness and death from diseases caused by contaminated food are a constant threat to public health and a significant impediment to socioeconomic development worldwide. It remains a major public health challenge regardless of being underreported [2].

The causes of foodborne illness include viruses, bacteria, parasites, toxins, and metals. The symptoms of foodborne illness range from mild gastroenteritis to life-threatening neurologic, hepatic, and renal syndromes. The main microorganisms responsible the FBDO are *Salmonella, Staphylococcus aureus, Clostridium perfringens, Bacillus cereus,* and *Campylobacter* [2,3]. According to the World Health Organization’s estimates in 2010, there would be 600 million cases of foodborne illness per year with more than 400,000 deaths [4]. Data released by the Centers for Disease Control and Prevention’s (CDC) Foodborne Diseases Active Surveillance Network (FoodNet) for 2016 demonstrated that the United States continues to make little progress in driving down the rates of infection by bacteria commonly transmitted through food [5]. In addition, in 2016, the CDC estimated the number of foodborne illness at 48 million cases annually with 128,000 hospitalizations and 3000 deaths [5,6].

In Morocco, an average of 100 FBDO episodes are reported annually, corresponding to 1500 cases notified by all provinces and regions of the kingdom. However, the laboratory confirmation rate remains very low, not exceeding the 10% threshold. In fact, according to the Department of Epidemiology and Disease Control (DEDC), during the period 2007 to 2017, 13,778 cases of FBDO were identified, of which 57.1% of the households were declared in a family environment, and 42.9% of these outbreaks were reported in communities [7].

Surveillance of foodborne illness is complicated by several factors including underreporting. Although a foodborne illness can be severe or even fatal, milder cases are often not detected through routine surveillance [8,9]. Many pathogens transmitted through food are also spread through water or from person to person, thus obscuring the role of foodborne transmission. Finally, some proportion of foodborne illness is caused by pathogens or agents that have not yet been identified and thus cannot be diagnosed. The importance of this final factor cannot be overstated. Many of the pathogens of greatest concern today (eg, *Campylobacter jejuni*, *Escherichia coli* O157:H7, *Listeria monocytogenes*, and others such as *Cyclospora cayetanensis* and Norovirus) were not recognized as causes of foodborne illness just decades ago [10]. Any episode of FBDO should be considered as an emergency and should be investigated immediately. The purpose of the investigation of FBDO was to avoid any extension and to prevent recurrences contributing to food security [6,10].

On Sunday, June 18, 2017, at 5 am, 30 students at the training institute in Rabat, who a day before had attended the Ramadan buffet in the canteen, exhibited the following gastrointestinal symptoms: diarrhea, nausea, vomiting, abdominal cramps, fever, and dizziness. Given the high number of affected cases and the coincidence of this crisis with the period of school end-of-year, this situation triggered a state of health emergency. At 9 am, 43 students among the 392 registered in this institute were admitted to the emergency room of the University Hospital in Rabat with symptoms of gastroenteritis. The director of the hospital quickly reported those cases to the regional health director of Rabat-Salé-Kenitra who alerted the Provincial Epidemiology Unit.

Owing to the large number of cases identified, similarities, commonality and location, the symptoms, and the occurrence context, an FBDO was suspected and an outbreak investigation team from the Field Epidemiology Training Program (FETP) and DEDC was immediately requested to initiate the outbreak investigation.

The investigation aimed to confirm the FBDO and to identify the source(s) of contamination and the causal agent to implement control measures and prevent further cases. A case control study was performed to identify specific risk factors associated with the occurrence of the foodborne illness outbreak in that institution.

## Methods

### Epidemiological Investigation

The outbreak investigation team, including 2 FETP fellows, visited the training institute in Rabat to collect all data related to the FBDO. The list of foods items in the menu of the last 3 days were recorded from the subcontracting company along with the list of persons admitted to the hospital. A structured questionnaire was developed to collect information about the food consumption and sociodemographic and clinical data of patients. An Excel sheet was used to develop the epidemic curve. All the necessary authorizations were obtained from the DEDC director and the regional health director of Rabat-Salé-Kenitra.

### Laboratory Investigation

Following notification of the outbreak, stool samples were collected for bacteriological analysis from 2 students admitted at the emergency department of the hospital. Meanwhile, bacteriological sampling was recommended for all restaurant staff to identify the presence of possible healthy carriers, especially for *Staphylococcus*. Food sampling was performed on the leftovers obtained from the institute’s restaurant. Food samples were sent to the National Reference Laboratory of the National Hygiene Institute and tested for *Salmonella, Shigella*, pathogenic *E coli*, *Campylobacter, Bacillus cereus, Clostridium, Yersinia enterolitica*, and *S aureus* and its toxin. A bacteriological analysis was done for the following items: chicken briwates, pastries, turkey steak, minced meat, madeleine, harcha, meloui, orange juice, and yogurt.

### Environmental Investigation

According to the general principles of food hygiene [11], an environmental assessment was undertaken by a multidisciplinary team, and the caterer’s premises were inspected to identify the conditions that may have contributed to the occurrence of the outbreak. The following critical points were inspected: the kitchen, the storage area, the sanitary rooms, the hot chain, the cold chain, and the catering area.

### Analytical Phase—Case-Control Study

We conducted a case-control study. Cases and controls were selected in a ratio of 1:1. We defined a case as any member of the training institute who attended the Ramadan buffet in the institute’s restaurant and who had presented, in the weekend of June 16 to 20, 2017, symptoms of diarrhea or vomiting with at least one of the following signs: abdominal pain, fever, headache, nausea, and dizziness. A control was defined as anyone who attended the Ramadan buffet in the institute’s restaurant but had not presented any symptoms from June 16 to 20, 2017.

### Data Analysis

A descriptive analysis was conducted on the basis of person, time, and place. Results have been presented using means or medians for quantitative variables and percentages for qualitative variables. An epidemic curve has been drawn with daily time steps and a 2-hour time interval. We also analyzed the distribution of onset of symptoms and demographic characteristics. A univariate analysis was performed to compare characteristics and exposure of cases and controls using the chi-square or Fisher Exact tests for categorical variables and *t* test or Wilcoxon test for continuous variables. Odds ratio (OR) and 95% CI were calculated for each exposure variable of interest (food items) and a *P* value of <.05 was considered statistically significant. A multivariate analysis was performed by using logistic regression. Data were collected and analyzed using Epi Info7 (CDC).

## Results

### Epidemiological Investigation

We identified 50 patients who consumed the suspected meal in the institute’s restaurant. Of those, 43 were males (86%) with a median age of 21 years (range 20-27 years) and an average of 21.86 (SD 1.4) years. The most frequent symptoms were diarrhea (50/50, 100%) and abdominal pain (49/50, 98%), followed by nausea (26/50, 52%), fever (18/50, 36%), dizziness (9/50, 18%), and vomiting (8/50, 16%). Bloody diarrhea was reported in 2 cases. A total of 43 students were transferred to the hospital, and case patients had complete resolution of symptoms with no hospital admission and no death.

The control population consisted of 31 men (62%) and 19 women (38%) with men to women sex ratio of 1.63. Among 50 controls, the median age was 22 years (range 18-47 years) and an average of 23.8 (SD 5.0) years.

Multimedia Appendix 1 shows the epidemic curve. The first 2 cases presented symptoms between 10 pm and 12 pm on June 17, and the number of cases peaked early in the morning on June 18, and the last reported illness onset was in the same day. The average incubation period was estimated as 9 hours.

The only common exposures shared by all case patients were food from the institute’s restaurant during a Ramadan dinner. On the basis of this information, the hypothesis was that the consumption of contaminated food from the institute’s restaurant was the source of the outbreak.

### Laboratory Investigation

The stool results of 2 patients were negative. *Staphylococcus aureus* and *E coli* 0157 H7 were isolated from food samples of briwates at levels exceeding the acceptable thresholds. In addition, coliform testing identified briwates contamination with coliforms. There was no result from testing among food handlers.

### Environmental Investigation

The inspection of the kitchen premises by the investigation team revealed the following:

- Defects in the design and maintenance of the restaurant premises: cracks in the ground with water infiltration.
- Unsatisfactory hygiene conditions with lack of hand washing and hand drying.
- Poor condition of the cold room, with a nonfunctional temperature indicator.
- Poor state of the laundry room and utensils stored in inadequate conditions.
- Presence of vectors in the kitchen (roaches and mosquitoes).

### Analytical Phase—Case-Control Study

Table 1 shows the food items consumed by cases and controls, a day before the onset of illness. In the univariate analysis, the exposure that was highly associated with the diseases was eating briwates (OR 14.2, 95% CI 5.05-40.04). In the multivariate analysis, the briwates eaten at dinner a day before remained strongly associated with the illness (OR 56.71, 95% CI 12.11-265.65; Table 2). Meanwhile, eating harcha and meloui appeared as protective factors with an adjusted OR of 0.06 and 0.14, respectively. Among briwates consumers, a significant association was noted between the occurrence of illness and meloui and harcha concomitant consumption (Table 3).

**Table 1:** Food items associated with the illness during a foodborne outbreak in a training institute in Rabat on June 18, 2017.

Food item	Cases, n (%)	Controls, n (%)	Odds ratio (95% CI)	*P* value
Briwates^a^	44 (88)	17 (34)	14.2 (5.05- 40.04)	<.001
Jam	2 (4)	8 (16)	0.22 (0.04-1.08)	.09
Cheese	26 (52)	41 (82)	0.23 (0.09-0.59)	.01
Harcha^b^	9 (18)	23 (46)	0.25 (0.10-0.64)	.01
Milk	13 (26)	20 (40)	0.52 (0.22-1.23)	.13
Madelaine	12 (24)	25 (50)	0.31 (0.13-0.74)	.01
Meloui^c^	8 (16)	19 (38)	0.3 (0.11-0.77)	.01
Eggs	27 (54)	30 (60)	0.78 (0.35-1.73)	.54
Bread	4 (8)	2 (4)	2.08 (0.36-11.95)	.67
Milk products	2 (4)	8 (16)	0.22 (0.04-1.08)	.09
Seffa^d^	8 (16)	6 (12)	1.39 (0.44-4.36)	.56
Turkey steak	8 (16)	3 (6)	2.92 (0.72-11.74)	.19
Minced meat	4 (8)	11 (22)	0.30 (0.09-1.04)	.09
Pastry	13 (26)	14 (28)	0.90 (0.37-2.18)	.82

^a^A traditional food prepared with chicken mixed with condiments, vermicelli, chopped onions, and eggs and manually wrapped by hand in a sheet of brick.

^b^A cake prepared with semolina, butter, and milk or water.

^c^A traditional pancake.

^d^A sweet couscous made with vermicelli, cinnamon, and almonds.

**Table 2:** Adjusted odds ratios for food items associated with the illness during a foodborne outbreak in a training institute in Rabat on June 18, 2017

Food item	Adjusted odds ratio (95% CI)	*P* value
Briwates^a^	56.71 (12.11-265.65)	<.001
Harcha^b^	0.06 (0.01-0.27)	<.001
Meloui^c^	0.14 (0.03-0.58)	.01

^a^A traditional food prepared with chicken mixed with condiments, vermicelli, chopped onions, and eggs and manually wrapped by hand in a sheet of brick.

^b^A cake prepared with semolina, butter, and milk or water.

^c^A traditional pancake.

**Table 3:** Association between the illness and consumption of meloui and harcha among the 61 consumers of briwates during a foodborne outbreak in a training institute in Rabat on June 18, 2017.

Food item	Cases (N=44), n (%)	Controls (N=17), n (%)	Odds ratio (95% CI)	*P* value
Harcha^a^	8 (18)	14 (82)	0.04 (0.01-0.2)	<.001
Meloui^b^	8 (18)	8 (50)	0.2 (0.06-0.7)	.01

^a^A cake prepared with semolina, butter, and milk or water.

^b^A traditional pancake.

## Discussion

### Principal Findings

A food poisoning outbreak occurred among participants at the Ramadan buffet in the canteen in a training institute in Rabat. This is consistent with the definition of a food poisoning outbreak [4,5]. Our results indicated that there was a strong association between patients’ status and eating briwates served at the Ramadan buffet on June 17. A total of 50 students had onset of food poisoning symptoms ranging from 2 hours to 17 hours after consuming a food item, consistent with the usual incubation period of foodborne illness related to *S aureus*, which is usually from 1 hour to 6 hours [12].

In our study, most case patients had minor symptoms; only 2 patients had bloody diarrhea. Symptoms had resolved within 1 hour after admission for the 43 patients who exhibited symptoms and visited the emergency department. This is in line with *Staphylococcus* food poisoning with a very rapid onset, usually within few hours after ingestion of contaminated food. In addition, only an estimated 10% of patients visited the hospital [13].

The epidemic curve suggests a significant point-source foodborne outbreak. *S aureus* and *E coli* 0157 H7 were isolated from food samples of briwates. The mixture of these microorganisms could have been responsible for this outbreak. However, the microbiological tests of collected stool samples from 2 cases were negative.

It should be noted that during the preparation of the briwates, the chicken is cut into small pieces and mixed with condiments, vermicelli, chopped onions, and eggs and then manually shaped and wrapped by hand in a sheet of brick in triangular form. It is stored at room temperature before being fried and placed in a cool place. The meals served on the menu of June 17, 2017, had been prepared the day before by several cooks at the caterer’s premises. It was not possible to know whether the farce of briwates was kept at night or not before being packed in the brick sheets. It is commonly known that FBDOs in institutional settings, where food prepared several hours before it has been served, are frequent and several micro-organisms are implicated, such as *E coli*, *S aureus*, *Salmonella species,* or even *C jejuni* and viruses [14].

It is demonstrated that foods requiring several manipulations during their preparation are frequently exposed to contamination by *Staphylococcus* and *E coli,* and the food handlers are often the main source of transmission [12,14].

The literature surrounding staphylococcal food poisoning outbreak publications is rare because of it being underreported. Food poisoning is a short-term illness and usually results in full recovery; doctors do not take it very seriously, especially when the outbreak affects only a few people [8,9,15]^.^

In Morocco, in 2016, the estimated incidence rate of foodborne disease was 4/100,000. Compared with other close areas, this rate was varying between the extremes of European values observed in Europe: 0.06/100,000 in Greece and 8.98/100,000 in Malta [7,16].

Few reports on foodborne illness outbreaks were published in Morocco. Over the last 6 years, 2 food poisoning outbreaks were reported in training institutes in almost similar circumstances. The first foodborne illness occurred in a training institute in Agadir, reported in 2011. That outbreak investigation indicated that there was an association between gastrointestinal illness and the consumption of hamburger and mayonnaise. Confirmed isolates of *Salmonella* unspecified were detected in the food items [17]. The second occurred in 2016 in a training institute in Rabat. It was related to the consumption of hamburger and milk semolina. *Salmonella* was isolated in hamburger, and *E coli*, in milk semolina and hamburger [18]. These 2 episodes led to the observation of 59 and 64 patients, respectively [17,18].

The average number of persons per outbreak in Moroccan published reports (58 persons per outbreak) was higher than that estimated from data reported by the European Food Safety Authority in 2016 (3.6 persons per outbreak). However, our estimation is limited only to 3 published reports, including this one.

The proportion of patients admitted in emergency rooms as a consequence of a foodborne disease in our study (86%) was higher than that reported in 2011 outbreak.

Among the strengths of our investigation is the prompt notification of the outbreak to the health authorities that facilitated rapid implementation of the first response actions. In addition to that, there was a quick case follow-up and sealing of the available food samples by environmental health inspectors. An active investigation of cases was conducted, and a face-to-face interview took place by applying a standardized questionnaire. These activities limited information biases related to the self-administered questionnaire. Finally, the collaborative work of the investigation team members, including epidemiologists, laboratory technicians, and environmental health inspectors, allowed for the rapid identification of the source of contamination.

There is a need for accurate data and strong evidence on the type of vehicles, causative agent, and the diversity of the isolated strains during an FBDO investigation. This study illustrates a dual-pathogen–related outbreak, which is not unusually reported in many foodborne outbreak investigations elsewhere [19].

One of the limitation of this study is that the enterotoxin was unknown although that the microbiological testing of food samples identified Staphylococcus aureus.

### Conclusions

In conclusion, there was evidence that the pathogens responsible for the food poisoning associated with the consumption of contaminated briwates were *S aureus* and *E coli*. The study illustrates the impact of gaps in the food handler control program, especially the lack of regard for hygiene best practices in collective catering. To reduce food contamination, continuous training on hygiene best practices, such as hand washing and use of gloves and protective clothing in kitchen areas, should be implemented. More attention should be paid by food handlers to ensure hand hygiene practices are followed to prevent a foodborne outbreak.

## Appendix

Multimedia Appendix 1 Epidemic curve of a foodborne outbreak in a training institute in Rabat on June 18, 2017.

## Abbreviations

CDC: Centers for Disease Control and Prevention
DEDC: Department of Epidemiology and Disease Control
FBDO: foodborne disease outbreak
FETP: Field Epidemiology Training Program
OR: odds ratio
