# Supplementary figures and images for: Outbreak Investigation of a Multipathogen Foodborne Disease in a Training Institute in Rabat, Morocco: Case-Control Study
Source: JMIR Public Health Surveill. 2019 Sep 25;5(3):e14227. doi: 10.2196/14227 (PMC6785723; doi:10.2196/14227)

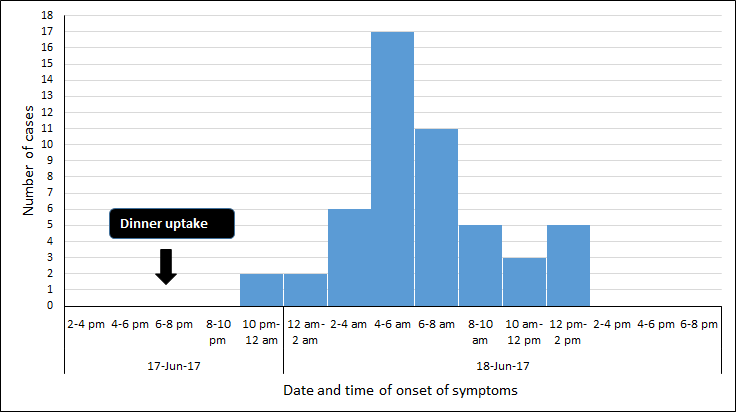

Supplement: Multimedia Appendix 1 [file publichealth_v5i3e14227_app1.png]
